# Promotion of healthy living environments in communities: a digital planning tool for local public health actors

**DOI:** 10.1093/eurpub/ckac129.445

**Published:** 2022-10-25

**Authors:** A Busskamp

**Affiliations:** Department for Cross Sectional Research, Federal Centre for Health Education Germany, Cologne, Germany

## Abstract

In their function as overarching settings, communities have a controlling role and political competence for the development and implementation of healthy living environments in Europe. However, the establishment of health-promoting structures is often hampered by a lack of resources and know-how, and decisions in practice are mostly based on personal experiences or standard procedures, which are often not in line with scientific findings and evidence-informed decision making. In the area of physical activity and older people, a digital planning tool (“Impulsgeber Bewegungsförderung”) compromising tools for needs assessment, interventions and information on physical activity, process management, legal issues and funding is being built to support the implementation of practices by local public health actors. This presentation will introduce the digital planning tool and its process evaluation. A mixed-method approach was applied to answer the question of whether and how the application can succeed in community settings and which additional needs of local actors exist. Initial steps of the recruited socio-economically disadvantaged model regions were setting up local steering committees and needs assessment using the tool. The model regions confirmed the usefulness of the tool in their day-to-day work, but used it with varying frequency. Among others, the tool helps to raise awareness among key actors, to reflect on their own work, or to identify funding sources. Wider use of the digital tool may lead to more evidence-informed actions in health promotion and prevention. The evaluation of the digital planning tool in a small area of health promotion will provide initial insights into how to support the implementation of activity-promoting living environments on a local level.

